# Regeneration of Critical‐Sized Mandibular Defects Using 3D‐Printed Composite Scaffolds: A Quantitative Evaluation of New Bone Formation in In Vivo Studies

**DOI:** 10.1002/adhm.202300128

**Published:** 2023-05-15

**Authors:** Sophia Dalfino, Paolo Savadori, Marco Piazzoni, Stephen Thaddeus Connelly, Aldo Bruno Giannì, Massimo Del Fabbro, Gianluca Martino Tartaglia, Lorenzo Moroni

**Affiliations:** ^1^ Department of Biomedical Surgical and Dental Sciences Università degli Studi di Milano Milano 20122 Italy; ^2^ Complex Tissue Regeneration Department MERLN Institute for Technology Inspired Regenerative Medicine Maastricht 6229 ER The Netherlands; ^3^ Fondazione IRCCS Ca' Granda Ospedale Maggiore Policlinico Milano 20122 Italy; ^4^ Department of Physics Università degli Studi di Milano Milano 20133 Italy; ^5^ Department of Oral & Maxillofacial Surgery University of California San Francisco 4150 Clement St San Francisco CA 94121 USA

**Keywords:** 3D printing, bone tissue engineering, composites, mandibles, maxillofacial defects, polymers

## Abstract

Mandibular tissue engineering aims to develop synthetic substitutes for the regeneration of critical size defects (CSD) caused by a variety of events, including tumor surgery and post‐traumatic resections. Currently, the gold standard clinical treatment of mandibular resections (i.e., autologous fibular flap) has many drawbacks, driving research efforts toward scaffold design and fabrication by additive manufacturing (AM) techniques. Once implanted, the scaffold acts as a support for native tissue and facilitates processes that contribute to its regeneration, such as cells infiltration, matrix deposition and angiogenesis. However, to fulfil these functions, scaffolds must provide bioactivity by mimicking natural properties of the mandible in terms of structure, composition and mechanical behavior. This review aims to present the state of the art of scaffolds made with AM techniques that are specifically employed in mandibular tissue engineering applications. Biomaterials chemical composition and scaffold structural properties are deeply discussed, along with strategies to promote osteogenesis (i.e., delivery of biomolecules, incorporation of stem cells, and approaches to induce vascularization in the constructs). Finally, a comparison of in vivo studies is made by taking into consideration the amount of new bone formation (NB), the CSD dimensions, and the animal model.

## Graphical abstract

This review depicts the state of the art, in the last decade, on the regeneration of critical‐sized mandibular defects through composite or polymeric AM scaffolds. In particular, biomaterials, scaffolds structural properties, biomolecules and cellular components, along with vascularization strategies are correlated to NB data obtained from in vivo studies on small and large size animal models.

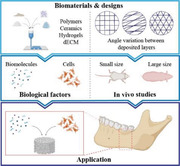

## 1 Introduction

Mandibular bone defects may be attributed to different causes. Small defects commonly originate from tooth extraction or minor traumas, while larger ones result from severe traumatic injuries, degenerative diseases, congenital disorders and tumor resections.^[^ ^1^ ^]^ If the dimensions of the defects are below a critical size, the bone tissue can restore the damage through physiological healing processes.^[^ ^2^ ^]^ Anyhow, to further boost the regeneration of small defects, some commercial products are already available, such as Boneceramic (a trademark of Straumann), Grafton DBM (a trademark of Biohorizons) and Bonalive, which are granule‐based synthetic materials. On the contrary, when a defect exceeds a critical size , the body is not able to bridge the gap between the two bone ends and eventually fibrotic tissue is deposited instead of mineralized healthy bone tissue.^[^ ^3^ ^]^ A critical size defect (CSD) was first defined by Schmitz & Hollinger (1986) as “the smallest size intra‐osseous wound in a particular bone and species that will not heal spontaneously during the lifetime of the animal.”^[^ ^4^ ^]^ This definition was later modified as follow: “the size of a defect that will not heal over the duration of the study”, because most studies have limited duration and do not extend the lifespan of the animal.^[^ ^5^ ^]^

The maxillofacial region has a complex anatomy and is the headquarters of several functions such as chewing, talking, swallowing and breathing, which induce constant stress in the mandibular bone. Moreover, this tissue presents peculiarities compared to most long bones in the human skeleton. Indeed, the mandible is known to have a fast‐remodelling kinetics and an embryonic development similar to craniofacial bones. Since it originates from the neural crest cells of the neuroectoderm germ layer, it undergoes intramembranous rather than endochondral ossification. Lastly, stem cells derived from mandibular bone marrow have been shown to exhibit higher osteogenic potential than those present in other skeletal bones.^[^ ^6^ ^]^ For all of these reasons, mandible regeneration needs to be specifically addressed, as tissue engineering strategies designed for long bone regeneration cannot be directly translated to it.

Current bone reconstruction strategies are based on filling defects with bone autografts, allografts, or xenografts that are able to support healing through three important processes: osteoinduction (i.e., differentiation of progenitor cells into osteoblasts), osteoconduction (i.e., bone tissue growth), and osseointegration (i.e., graft integration in the surrounding native bone).^[^ ^7^, ^8^, ^9^ ^]^ Specifically for mandibular bone reconstruction, the gold standard is the fibular flap autograft, which was first introduced by Hidalgo in 1989.^[^ ^10^ ^]^ This technique consists in two invasive surgical procedures; a first resection of a portion of the fibula and its successive fixation within the mandibular defect using metallic plates.^[^ ^10^, ^11^ ^]^ In this regard, load‐bearing reconstruction plates are required to ensure mechanical support especially during the early days of recovery. Nowadays, the plates can be hand‐bent or specifically shaped on the patient CSD, thanks to modern computer‐aided design (CAD) and computer‐aided manufacturing (CAM) technologies. Along with other autologous bone grafts, the fibular flap cannot cause immunorejection related problems and contains natural biological molecules, such as bone morphogenic proteins (BMPs) and platelet‐derived growth factor (PDGF), which ensure osteogenic pathways ignition.^[^ ^12^, ^13^ ^]^ Nonetheless, despite advances in the surgical procedure, it remains an invasive technique requiring at least two surgeries, which can result in patient discomfort and potential morbidity. Furthermore, the amount of harvested material may be insufficient when dealing with excessively large CSDs, and implant integration with the native bone can fail.^[^ ^9^ ^]^ Finally, post‐operative cares are also important to ensure a complete recovery from the surgery, preventing suboptimal tissue performance and altered facial physiognomy, thus compromising patient's quality of life.^[^ ^14^, ^15^ ^]^

**Figure 1:**
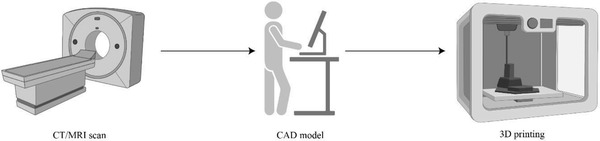
Steps of the scaffold printing process. Patient's defect is obtained through CT or MRI images; a virtual 3D model of the scaffold is created with CAD software; the scaffold is manufactured using a 3D printer. Created with BioRender.com.

Limitations of the current gold standard clinical procedure is driving mandibular bone tissue engineering (BTE) toward the use of synthetic substitutes as alternatives to biological grafts. In this context, additive manufacturing (AM) is considered the most promising technique in regenerative medicine to fabricate advanced materials as scaffolds.^[^ ^1^ ^]^ The reasons are multiple: rapid prototyping, high repeatability, low production costs, free‐form fabrication, and patient‐specific fidelity.^[^ ^16^ ^]^ This last point can be addressed by obtaining defect anatomy from an imaging source [i.e., computed tomography (CT), magnetic resonance (MRI)], which is then converted into a printable object model through CAD and slicer software (**Figure** **1**).

Scaffolds produced via AM should mimic the mandibular tissue in terms of macro‐ and microstructure, composition and function. Indeed, a scaffold has to be porous with interconnected pores to favor mass transport of oxygen and nutrients into the inner core of the construct and to allow homogeneous cell colonization.^[^ ^17^, ^18^, ^19^ ^]^ Higher values of porosity and pore diameter, in general, correlate with greater bone formation and superior vascularization.^[^ ^20^, ^21^ ^]^

An advantage of using AM techniques is the possibility to print several classes of biomaterials.^[^ ^22^, ^23^ ^]^ However, those employed in mandibular BTE are essentially limited to calcium phosphate ceramics and synthetic polymers. The first category includes hydroxyapatite (HA) and *β*‐tricalcium phosphates (*β*‐TCP), whereas the second one, poly(glycolic acid) (PGA), poly(lactic acid) (PLA), poly(lactide‐co‐glycolic acid) (PLGA), poly(caprolactone) (PCL) and photocurable polymers (i.e., resins). Both ceramics and polymers can be printed with different AM techniques such as vat photopolymerization [i.e., stereolithography (SLA) and digital light processing (DLP)], selective laser sintering (SLS) and fused deposition modelling (FDM).^[^ ^24^ ^]^

In addition, to tune biomaterials chemical composition and structure parameters, many studies demonstrate that including biological elements in the construct can boost mandibular bone reconstruction much further.^[^ ^25^ ^]^ Of marked relevance there are osteoinductive bioactive molecules (e.g., BMPs) and growth factors that promote the healing process (e.g., PDGF). They can be encapsulated in specific delivery systems (e.g., hydrogels) to achieve simultaneously spatial‐ and time‐controlled release from the scaffold, and prevent heterotopic bone formation due to high dose‐related side effects.^[^ ^25^ ^]^ Cells like mesenchymal stromal cells (MSC) and endothelial cells can also be added to stimulate new bone formation (NB) and vascularization within the defect area.^[^ ^26^ ^]^ Additionally, the scaffold can be prefabricated in a living bioreactor (i.e., a heavily vascularized tissue of the body) prior implantation in the mandibular bone, to establish a mature capillary network.^[^ ^27^ ^]^

This review aims to illustrate the state of the art in the specific field of mandibular bone CSDs regeneration using polymeric or composite scaffolds fabricated through AM techniques. To the best of our knowledge, most of the reviews found in literature address the bone regeneration topic focusing on long bones; although, as previously discussed, the mandibular bone presents peculiarities that make its reconstruction a more challenging clinical reality. Moreover, this review differs from others addressing the field of mandibular BTE. Park et al.^[^ ^28^ ^]^ recently reported an analysis of the on‐demand 3D bio‐printing methods for the reconstruction of mandibular defects, whereas Nyirjesy et al.^[^ ^29^ ^]^ investigated the history and evolution of 3D printing for head and neck oncologic surgery and bone reconstruction. The main goal of our work is instead to highlight the best scaffold features to ensure optimal mandibular bone regeneration. In particular, we will discuss materials chemical composition, AM techniques, structural and design parameters, bioactive molecules, vascularization strategies, and cell types. At the end, mandibular BTE strategies will be quantitatively evaluated in terms of NB data obtained from in vivo studies, thus facilitating an objective and straightforward comparison among different experimental approaches, CSD dimensions and animal models.

## 2 Research Strategy

In order to find articles pertinent to this review, PubMed and Scopus electronic databases were searched using the following search strategy: (scaffold) AND (mandible[Title/abstract] OR jaw[Title/abstract] OR mandibular[Title/abstract]) AND (3d printing OR 3d printed OR three dimensional printed OR three dimensional printing OR additive manufacturing) AND (polymer OR composite OR polymeric). Furthermore, only articles published in the last decade (from 2012 to 2022) in English language were considered. No limitation regarding study design was set. A total of 23 articles were selected (**Table** **1**).

**Table 1:** Overview of reviewed articles. Column headers represent: i) material chemical composition, ii) scaffold structural properties (i.e., pore size and overall porosity), iii) biologically active molecules, iv) cells added in the constructs, v) size of the animal model used for in vivo experiments, and vi) main results in terms of new bone formation

Materials	Structure	Biomolecules	Cells	Animal model	Results	Ref.
HA/PLGA	Ø = 480–520 µm	–	–	Small	BV/TV ≈ 25% MV/TV ≈ 45% at 8 weeks	[30]
LAY‐FOMM 60	Ø = 750 µm (macropores) Ø = 0.2–20 µm (micropores)	–	DPSCs	Small	BV/TV = 30.26 ± 9.46%	[31]
PLGA/nHA	Porosity = 74% Ø = 430 µm	rhBMP‐2	–	Small	BV/TV ≈ 25% at 8 weeks	[32]
PCL/TCP	NA	–	hAFSCs	–	Cell viability = 91 ± 2% after 1 day in the printed bone	[33]
*β*‐TCP/PCL	Ø_1_ = 5–40 µm Ø_2_ = 7–300 µm	–	pBMPCs	Big	Bone PSA = 22.11 ± 22.45	[34]
PCL/TCP/bdECM	NA	–	ADSCs	Big	NBV = 372.32 HU after 8 weeks	[35]
PCL/*β*‐TCP	Porosity = 57% Ø_1_ = 300 µm Ø_2_ = 600 µm	–	–	Big	BV/TV ≈ 13% after 12 weeks	[36]
*β*‐TCP	Ø = 330 µm	Dipyridamole	–	Small	BV/TV ≈ 25% after 8 weeks	[37]
PLA	Ø = 50–300 µm	nHA, carbon nanotube	C3H/10T1/2	–	Cell viability = 150% from MTT assay at 7 days	[38]
PCL/*β*‐TCP	Porosity = 50% Ø = 500 µm	rhBMP 2	MSCs	Big	BV/TV = 48.443 ± 0.25% after 12 weeks	[39]
PCL/*β*‐TCP	NA	–	TMSCs	Small	BV/TV = 57.44% after 12 weeks	[40]
PCL/*β*‐TCP/hydrogel	NA	Resveratrol (RSV), strontium ranelate (SrRn)	MSCs, osteoclasts, HUVECs	Small	BV/TV ≈ 25% after 8 weeks	[41]
PLGA/*β*‐TCP	Porosity = 63.7% Ø = 358 µm	rhBMP‐2	–	Big	BV/TV ≈ 20% after 3 months	[42]
PCL/HA	Porosity = 83.3% Ø = 470 µm	–	MC3T3‐E1	–	Cell proliferation assessed at 7 days	[43]
Magnesium‐substituted calcium silicate, scaffolds	Porosity = 58% Ø = 480 µm Ø = 600 µm Ø = 720 µm	Ions	–	Small	BV/TV ≈ 25% at 12 weeks	[44]
HA/TCP	NA	rhBMP‐2	–	Big	BV/TV ≈ 40% after 12 weeks	[45]
*β*‐TCP	Porosity = 64%	–	–	Big	After 6 months CT data confirmed osseointegration	[46]
PCL/*β*‐TCP	Porosity = 57%	rhBMP‐2	MC3T3‐E1	Big	NBV = 10.08 ±2.48 mm^3^	[47]
PCL	Porosity = 20–80%	–	ADSCs	Small	In vivo assessment of vascularization after 7 days	[48]
PEEK	Porosity = 50% Ø = 750 µm	–	ADSCs	Small	BV/TV = 61.27% ± 8.24 at 20 weeks	[49]
PLA	Porosity = 85%	BMP‐2	–	Big	BV/TV = 25–32% after 3 months	[50]
PGLA/HA	Ø = 400 µm	–	–	Small	MV/TV = 29.88 ± 4.61% at 4 weeks	[51]
PCL/nHA	Porosity = 53.53%	–	–	Big	Histologic results confirmed angiogenesis and bone formation	[52]

NA = not available; BV/TV = bone volume/total volume; PSA = percentage surface area; HU = Hounsfield unit; NBV = new bone volume; MV/TV = mineral volume/total volume; DPSCs = Dental Pulp Stem Cells; hAFSCs = Human Amniotic Fluid Stem Cells; pBMPCs = Peripheral Blood Mononuclear Cells; ADSCs = Adipose Tissue‐derived Stem Cells; C3H/10T1/2 = cell line from C3H mouse embryo cells; MSCs = Mesenchymal Stem Cells; TMSCs = Tonsil‐derived Mesenchymal Stem Cells; HUVECs = Human umbilical vein endothelial cells; MC3T3‐E1 = immature osteoblast murine cell line.

## 3 Biomaterials for Mandibular Regeneration

**Figure 2:**
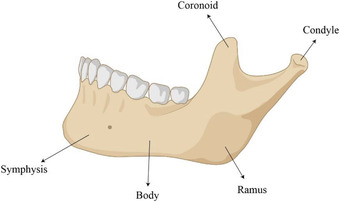
Anatomy of the mandible. Created with BioRender.com.

Similarly to long bones, the mandible can be defined as a composite material consisting of a mineral phase of calcium phosphates, an organic phase of collagen type I and non‐collagenous proteins, and water. The amount of each part may vary depending on the patient's age, location, gender, ethnicity, and medical conditions.^[^ ^53^ ^]^ Imamura et al. recently compared the micro‐structure and bone composition of human mandibular specimens, harvested from the symphysis and the ramus, to the tibia and ilium (**Figure** **2**).^[^ ^54^ ^]^

Using micro‐CT (mCT), they measured several parameters, such as the percentage fraction of the bone volume over the total volume (BV/TV%) and the bone mineral density (BMD), defined as the volume density of calcium hydroxyapatite (mg cm^−3^). Higher values of BV/TV% were revealed in the mandibular samples compared to those extracted from the tibia and ilium (**Table** **2**). These results suggested that different bone sites are characterized by compositional and structural differences. This feature should be considered for an accurate evaluation of the in vivo results.

**Table 2:** Comparison of BMD and BV/TV in four different sites[54]

	Symphysis	Ramus	Tibia	Ilium
BV/TV [%]	34.4 ± 13.7	30.1 ± 9.2	18.1 ± 6.7	22.0 ± 8.8
BMD (mg cm^−3^)	1030.9 ± 168.3	859.7 ± 130.5	813.1 ± 115.0	756.6 ± 112.3

To achieve good results, the ideal scaffold should be designed to mimic the microstructure and composition of natural bones as closely as possible, which involves selecting appropriate biomaterials. The most commonly used biomaterials for the production of scaffolds for mandibular tissue regeneration are synthetic polymers (e.g., PCL, PLA, and PLGA) and ceramics (TCP and HA) (Table 1). These two classes of materials can both be processed through AM techniques. Synthetic polymers are synthetized under controlled and reproducible conditions, offer good mechanical properties, and are generally biodegradable and biocompatible. Their degradation time varies from a few months to a few years, depending on the exact chemical composition and molecular weight. It is also possible to control the impurities present in the material, reducing the risk of toxicity and infection.^[^ ^55^ ^]^ PLA is a thermoplastic polyester that has been used for various medical implants, including bone screws, fixation devices and vascular grafts, due to its biocompatibility, degradability, high mechanical strength and low immunogenicity.^[^ ^18^, ^38^ ^]^ Manjunath et al. reported FDM‐fabricated PLA scaffolds, embedded with a PCL matrix to release biomolecules into the external environment.^[^ ^38^ ^]^ In addition, clinical‐grade PLA scaffolds coated with a polyelectrolyte were engineered to release BMP‐2 proteins.^[^ ^50^ ^]^ In both cases, the PLA scaffolds acted as the structural component, providing the shape of the anatomical part to be regenerated. PCL has been extensively studied for tissue engineering, due to its physico‐chemical properties, such as mechanical strength, prolonged biodegradation (almost three years for complete removal from the body for PCL with average molecular weight = 50 kDa) and biocompatibility.^[^ ^34^, ^38^, ^55^ ^]^ The high mechanical strength and slow degradation rate of PCL allow for load bearing properties during gradual formation of new bone tissue. This is an important property, since mandibular bone has to face high loads during the patient's life. Temple et al. and Zamani et al. confirmed the successful use of PCL alone to print structures to support mandible tissue regeneration.^[^ ^48^, ^56^ ^]^ PLGA is a synthetic copolymer of PLA and PGA. It has excellent degradability, which can be adjusted according to the ratio of PLA to PGA, and biocompatibility, and is widely used as a drug carrier material.^[^ ^32^ ^]^

Despite their good mechanical properties and biocompatibility, synthetic polymers usually have limited cell adhesion properties, and lack of bioactivity (i.e., the ability of a material to induce and accelerate the mineralization on its surface).^[^ ^57^ ^]^ Therefore, they can be combined with bioceramics that are generally osteoinductive and osteoconductive.^[^ ^55^, ^58^ ^]^ The most commonly used bioceramics for mandible regeneration are calcium phosphates, because they are already present in high amounts in the native bone tissue.^[^ ^18^ ^]^ These materials have excellent osteoinductivity and osseointegration properties because of their chemical composition.^[^ ^59^ ^]^ The release of Ca^2+^ and PO_4_^3−^ ions in the surrounding environment sure enough is a signal that guide cell migration, bone remodelling, and matrix mineralization.^[^ ^59^, ^60^ ^]^ Phosphate ceramics exhibit, in general, lower toughness compared to the natural cortical bone and this restricts their use to non‐load bearing applications.^[^ ^61^ ^]^ Nevertheless, it is worth noting that mechanical strength depends on particle size and shape, and it can be tuned by mixing different ceramics or performing treatment on the ceramic powders.^[^ ^61^, ^62^ ^]^ HA is the main inorganic component of bone tissue and for this reason has intrinsic excellent properties for mandibular BTE, like high biocompatibility, no cytotoxicity and long‐term biodegradability. This last feature can be also tuned by sintering temperature, porosity, and pore diameter.^[^ ^63^ ^]^ The optimal osteoconductive properties allow HA scaffolds to form a strong bond with the surrounding bone.^[^ ^18^ ^]^ HA has been shown to induce the proliferation and osteogenic differentiation of human bone marrow‐derived MSCs in vitro, as it is able to promote the expression of osteogenic growth factors, such as BMP and alkaline phosphatase (ALP).^[^ ^18^, ^30^, ^32^ ^]^ Therefore, Ciocca et al. functionalized PCL scaffolds with nHA to increase hydrophilicity and enhance cell adhesion and differentiation.^[^ ^52^ ^]^ Both Deng et al. and Chang et al. reported that combining HA and PLGA can increase the bioactivity of the polymer.^[^ ^30^, ^32^ ^]^ Interestingly, Chang et al. printed a hyperelastic biomaterial made of 90% of HA and 10% of PLGA to evaluate its osteoregenerative ability.^[^ ^51^ ^]^ Such a high amount of HA present in the composite material should enhance the bioactivity of the scaffold, slow down polymer degradation rate as well as the amount of by‐products derived from it. Another category of ceramics is *β*‐TCP, which has a faster resorption compared to HA.^[^ ^34^ ^]^ In addition, *β*‐TCP has a high affinity for BMP‐2, which is a key growth factor required for bone production. Because of these characteristics, *β*‐TCP can lead to a rapid bone formation straight after implantation, improving the regeneration of mandibular bone tissue. This is why it is often used in combination with polymers to create composite scaffolds.^[^ ^35^ ^]^ A composite scaffold based on PCL and *β*‐TCP was tested in several studies (**Table** **3**), with different ratios of the two components, showing promising results in different animal models. Composite scaffolds of 50/50 (%w/w) PCL/*β*‐TCP, pre‐seeded with porcine MSCs, induced a good depth of bone penetration in pig defects.^[^ ^34^ ^]^ PCL/*β*‐TCP composites with different relative compositions and pore sizes were also tested by Lee et al. in a beagle model, resulting in good bone formation (NBV = 30.50 ± 16.26 mm^3^) and regeneration of the critical mandibular defect.^[^ ^36^ ^]^

**Table 3:** Compositions of the composite scaffolds made with PCL and *β*‐TCP

PCL [% w/w]	*β*‐TCP [% w/w]	PCL:*β*‐TCP	Printing technology	Composite production	Ref.
50	50	1:1	Ink‐jet printer	–	[34]
70	30	2.3:1	Multi‐head deposition system	Melting and mixing	[36]
50	50	1:1	3D‐Bioplotter (FDM)	Melting and mixing	[39]
50	50	1:1	FDM	–	[40]
NA^a)^	NA^a)^	1:2	3D‐Bioplotter	Solvent method	[41]
70	30	2.3:1	Micro extrusion‐based 3D printer	Melting and mixing	[47]

^a)^ NA = not available.

Ceramic materials have also been used as fillers for photocurable polymers. For example, Dienel et al. reported the development of a cross‐linked network of methacrylate poly(trimethylene carbonate) (PTMC) and *β*‐TCP, processed by SLA, to fabricate a CSD‐specific composite scaffold, for mandibular reconstruction in a Mini Pig model. However, NB was not significantly higher than the one of the empty defects (negative control), meaning that the defect size was not critical.^[^ ^64^ ^]^

In addition to polymer‐ceramic, also ceramic‐ceramic blends were investigated. One study, for example, combined both HA and *β*‐TCP to obtain a slurry processed with DLP.^[^ ^45^ ^]^ Unfortunately, after 12 weeks the NB in the experimental group (scaffolds) was lower than the positive control group (Bio‐Oss).^[^ ^45^ ^]^ Similarly, Kim et al. developed a custom‐made HA/*β*‐TCP scaffold through DLP printing, and tested it in beagle dogs with a mandible defect. After 8 weeks of implantation the NB induced by the scaffold was not significantly higher compared to the control group and the non‐treated group (empty defect), because, once again, the defect size was not critical.^[^ ^65^ ^]^

Taken together, mandibular bone regeneration is still facing numerous issues due to the complexity of the native tissue environment to be reconstructed. Composite scaffolds, which combine the properties of polymers and ceramics, are attracting many research efforts. In fact, as reported in Table 1, the most used materials are thermoplastic polymers in combination with HA and TCP, as ceramic fillers. These materials are typically processed into scaffolds via extrusion‐based deposition systems (Table 3). Despite other classes of materials have been investigated, such as photocurable polymers, more in vivo studies with CSDs have to be performed in order to demonstrate the true potential of these materials. Lastly, as proposed by Qin et al., another emerging category of materials in the mandibular BTE field are bioactive glasses and inorganic ions (e.g., Mg^2+^), which are used to dope scaffolds to enhance osteogenic and angiogenic potential.^[^ ^44^ ^]^ These materials could be employed alone or in combination with polymers to create innovative composite materials for mandible regeneration.

## 4 Additive Manufacturing Techniques for Mandibular Regeneration

In mandibular BTE is crucial to reproduce the natural macro‐architecture of the tissue to ensure proper functionality and facial physiognomy. It seems evident that a patient‐specific approach is of paramount importance. This can be achieved through AM techniques that enable the fabrication of scaffolds layer by layer starting from patient CT scans. Moreover, AM offers the possibility to process various materials, accomplish complex geometries, and generate porous structures. In BTE, a wide variety of techniques are employed, and they can be categorized in seven classes: material jetting, binder jetting, vat photopolymerization (e.g., DLP, SLA), powder bed fusion [e.g., SLS, selective laser melting (SLM)], material extrusion [e.g., FDM, liquid deposition modeling (LDM), robocasting], and electrospinning.^[^ ^66^, ^67^ ^]^ However, for mandibular applications, only SLS, inkjet printing, FDM, LDM, robocasting, and DLP have mostly been employed (**Table** **4**).

**Table 4:** AM techniques and materials used for the fabrication of scaffolds for mandibular regeneration

AM category	AM technique	Materials	Ref.
Powder bed fusion	SLS	PEEK powder	[49]
Material jetting	Inkjet printing	PCL/*β*‐TCP composite	[34]
Material extrusion	LDM	HA/PLGA slurry at room temperature (RT)	[30]
	LDM	PCL/*β*‐TCP solution + hydrogel at RT	[41]
	LDM	PLGA/*β*‐TCP solution at RT	[42]
	LDM	PCL/HA solution at RT	[43]
	LDM	PGLA/HA solution	[51]
	FDM	Melted Lay‐Fomm	[31]
	FDM	Melted lyophilized PLGA/nHA composite	[32]
	FDM	Melted PCL/*β*‐TCP composite	[33, 35, 36, 39, 40, 47]
	FDM	Melted PLA	[38, 50]
	FDM	Melted *β*‐TCP/stearic acid composite	[46]
	FDM	Melted PCL	[48]
	Robocasting	Colloidal gel formulation of *β*‐TCP	[37, 51]
Vat photopolymerization	DLP	Magnesium‐substituted calcium silicate scaffolds (UV)	[44]
	DLP	HA/TCP (UV)	[45]

**Figure 3:**
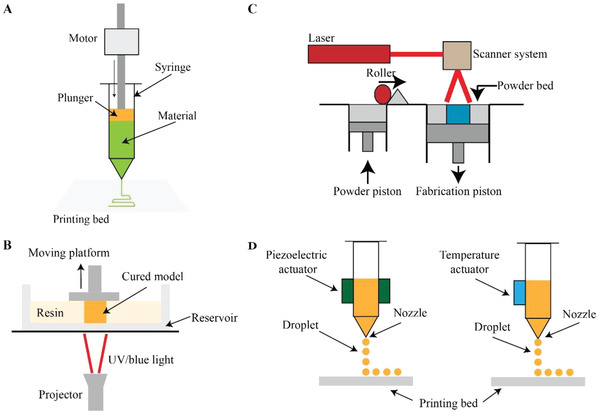
AM techniques used for mandibular BTE. A) Extrusion‐based system; B) DLP system; C) SLS process; D) Inkjet printing process.

LDM, FDM and robocasting are the most used techniques. They are extrusion‐based printing systems, in which a single material or a composite is extruded under pressure through a nozzle as a continuous filament (**Figure** **3**A).^[^ ^66^ ^]^ These techniques are compatible with a wide range of processable materials and have very low production costs. However, they are usually associated with limited resolution (≈300 µm), and the final parts can exhibit anisotropic behavior.^[^ ^22^, ^67^, ^68^ ^]^ FDM is based on the extrusion of melted materials; those that are mainly used for mandible reconstruction are composites formed by a polymeric (i.e., PCL, PLA, PLGA) and a ceramic part (i.e., TCP, HA). Since the melting process usually implies high temperatures, biological organic components (e.g., growth factors) need to be eventually incorporated at a later time. For instance, Lee et al. have successfully printed PCL/*β*‐TCP composite scaffolds at 120 °C, obtaining an interconnected structure with a porosity around 57%. After printing, the scaffolds were soaked in a collagen solution containing rhBMP‐2, to evaluate its effect on bone formation.^[^ ^36^ ^]^ Similarly, Lopez et al. have printed *β*‐TCP scaffolds with robocasting, starting from a colloidal ink of the ceramic powders in a water‐based solution. This AM technique required the scaffold to be sintered at high temperatures up to 1100 °C. After the post‐processing procedure, the scaffold was coated with a bioactive molecule of interest (i.e., dipyridamole).^[^ ^37^ ^]^ On the contrary, Zhang et al. have demonstrated the possibility to print a liquid PCL/*β*‐TCP composite solution at RT, incorporating small molecules during printing.^[^ ^41^ ^]^ In this way, the molecules have been found in the bulk of the fibers and not just on the surface. Indeed, recently LDM gained attention, because it allows the printing of viscous polymeric or composite solutions at low temperatures.^[^ ^69^ ^]^ Thus, it could be employed to incorporate biological organic factors (e.g., proteins) in the scaffold structure during printing without affecting their functionality.

One more AM category used for scaffold fabrication is vat photopolymerization. It involves the use of photosensitive liquid materials, called resins, that can be cured upon exposure to a specific light source, creating a 3D object. Among the vat photopolymerization techniques there are SLA and DLP. SLA exploits a focused laser beam to photopolymerize the material in a specific point; this technique guarantees high levels of detail and surface resolution (≈30–70 µm).^[^ ^68^, ^70^, ^71^ ^]^ DLP employs instead a blue or UV light from a projector to cure the resin into the desired shape (Figure 3B). Even though DLP can obtain objects with lower resolution (≈70–100 µm) than SLA, it benefits of faster processing speed, and extremely good surface quality.^[^ ^66^, ^68^, ^72^ ^]^ For example, Qin et al. have fabricated bioceramics scaffolds through DLP. Ceramic powders, composed of magnesium‐substituted calcium silicates, were mixed with a commercial resin and a photo‐initiator. Then, the material have been cured with UV light at a wavelength of 405 nm to create scaffolds with different pore dimensions.^[^ ^44^ ^]^ Also Ryu et al. have fabricated HA/TCP scaffolds through DLP. The powders have been mixed with a photo‐reactive ceramic resin, photo‐initiators, acrylic monomers, and dispersant. The final mixture was printed and cross‐linked with UV rays. In this case, the scaffolds required a post‐processing (sintering at 1250 °C).^[^ ^45^ ^]^ Despite the high resolutions that can be reached with vat photopolymerization techniques, there are some challenges that limit their translation into clinical application, such as the limited availability of biocompatible resins and photo‐initiators, degradation time of the scaffolds and high costs.^[^ ^66^, ^67^ ^]^

Two other less commonly used AM techniques are SLS and inkjet printing. SLS belongs to the powder bed fusion category, and relies on a laser energy source to locally sinter the material, usually in the form of powder (Figure 3C).^[^ ^23^ ^]^ Although scaffolds generated with this technique require post‐processing operations, SLS allows large and very complex structures to be produced at good resolution (≈500 µm).^[^ ^23^, ^66^, ^67^, ^68^ ^]^ An example is reported in the work of Roskies et al. A 3D scaffold of PEEK was manufactured with a CO_2_ laser source, obtaining a total porosity around 50% and a pore size of 730 µm.^[^ ^49^ ^]^

Lastly, Konopniki et al. have used inkjet printing to fabricate PCL/*β*‐TCP scaffolds. Inkjet printing consists in the extrusion of droplets of materials through a piezoelectric or thermal actuator (Figure 3D).^[^ ^73^ ^]^ The scaffold obtained had both macro‐ and micro‐porosity thanks to the drop‐by‐drop deposition method.^[^ ^34^ ^]^

To conclude, it is clear that every AM techniques have some advantages and disadvantages. However, they are certainly the most promising manufacturing methodology to replicate the mandibular tissue in terms of architecture and composition, in the direction of a more patient‐specific treatment.

## 5 Scaffold Structure and Design

**Figure 4:**
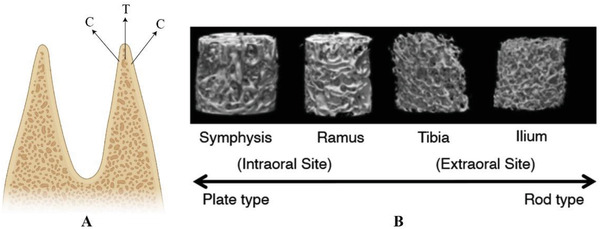
A) Mandibular bone structure: C = cortical bone layer, T = trabecular bone layer. Created with BioRender.com B) 3D morphological observations of the bone biopsy sample. Reproduced with permission.^[^ ^54^ ^]^ Copyright 2021, Elsevier.

In designing a scaffold it is crucial to take into account the structural and mechanical properties to promote the regeneration of a tissue. From a structural point of view, the bone tissue of the mandible is formed by two outer layers of cortical bone and a thick inner portion of trabecular bone (**Figure** **4**A). This structure has been confirmed by bone biopsy samples, harvested from the mandibular symphysis and the ramus.^[^ ^54^ ^]^ There are evident dissimilarities between mandible's trabeculae and those of other bones (e.g., tibia and ilium). Since different mandibular regions have to fulfil different functions and might present an inhomogeneous dentition state, intra‐variations within the mandible bone cancellous structure is also expected.^[^ ^43^ ^]^ Through mCT images (Figure 4B) it was demonstrated that plate‐like trabeculae were present in the symphysis and organized in different directions, creating a complex structure.^[^ ^54^ ^]^ In the ramus, the plate‐like trabeculae were wider and shallower than those found in the symphysis.

In mandibular BTE, the structural properties, in terms of pore shape, pore size and total porosity, influence cell differentiation, angiogenesis and bone growth, and determine to what extent the scaffold conformation resembles the native bone architecture. However, the determination of optimal scaffold pore size for craniomaxillofacial bone repair remains controversial. Several studies report a minimum pore size of 100 µm to achieve cell migration, and a pore size above 300 µm to achieve vascularization and bone formation.^[^ ^44^ ^]^ Moreover, interconnectivity of pores should be 100%, to provide space for cell migration and nutrient transport. This can be easily achieved with AM that allows fine fiber deposition control so to create open‐cell porous materials. Lastly, the total porosity should be between 70–80%. In fact, it was demonstrated that higher porosities (65–75%) led to higher in vivo bone formation and ALP activity than lower porosities (25%).^[^ ^20^ ^]^ Although an increase in porosity and pore size facilitates bone growth, it also affects the structural integrity of the scaffold. If porosity becomes too high, it can negatively influence the mechanical performance of the scaffold. For this reason, the upper limit of total porosity is usually kept around 80%. Therefore, it could be concluded that scaffolds should be fabricated with a pore size in the range of 300–900 µm and a porosity between 60% and 80%.

**Figure 5:**
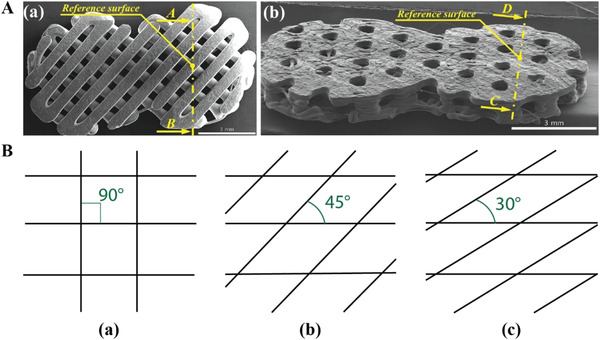
A) 0–90° pattern (a) and kagome structure (b). Reproduced with permission.^[^ ^76^ ^]^ Copyright 2019, Elsevier. B) Schematics of different fibers orientation: 0–90° (a), 0–45° (b), 0–30° (c).

Another important morphological parameter is the pore shape, which has been shown to have an impact on bone formation, both in vitro and in vivo.^[^ ^74^, ^75^ ^]^ All the reviewed studies (Table 1) have printed scaffolds using a 0–90° fiber orientation, resulting in square‐shaped pores, except for two of them, which proposed a different pattern. Interestingly, Lee et al. have compared the 0–90° pattern (grid‐structure scaffold) with a kagome structure (**Figure** **5**A) to improve the low mechanical properties of 3D printed PCL scaffolds, used to fill a calvarial defect.^[^ ^76^ ^]^ The kagome structure is a quarter‐cube honeycomb structure that creates a series of tetrahedra and truncated tetrahedra.^[^ ^77^ ^]^ The structures were designed to have a porosity of 50%. In mechanical tests, the kagome structure had shown to improve both ultimate compressive stiffness (UCS = 59.95 ± 2.91 MPa) and bending modulus (*E*_flex_ = 171.89 ± 17.75 MPa), compared to the grid structure (UCS = 42.73 ± 5.89 MPa and *E*_flex_ = 73.32 ± 12.89 MPa). Similar structures could be translated to mandibular bone application to improve the mechanical properties of the scaffolds. Liu et al. have printed three different models, at 90°, 45° and 30° (Figure 5B), with a total porosity between 80% and 90%, to compare the different structures in terms of mechanical properties, proliferation and osteogenic differentiation of MC3T3‐E1 cells seeded in the scaffolds.^[^ ^43^ ^]^ The mechanical properties were higher in the 0–90° scaffolds than in the other two architectures, with a compressive modulus (E) of 2.0 ± 0.15 MPa for PCL/HA scaffolds. The 0–90° scaffolds showed the highest E, while the 0–30° scaffolds showed the lowest one. Overall, the 0–90° fiber crossing angle structures (FCAS) significantly increased the compressive modulus compared to the 0–30° and 0–45° FCAS in all groups (*p* < 0.01). Furthermore, increased cell proliferation and ALP activity were observed for the 0–90° structure after seven days in the PCL/HA scaffolds in vitro.

The last important feature is the mechanical performance of the scaffolds, which should be similar to that of the native tissue in order to promote efficient scaffold osseointegration and support tissue functionality. The cortical bone tissue displays Young's modulus in the range of 15–20 GPa and UCS around 100–200 MPa, whereas the cancellous bone tissue has E in the range of 0.1–2 GPa and UCS around 2–20 MPa.^[^ ^8^ ^]^ By comparing these physiological values with scaffolds that have been reported so far in literature, it can be assessed that AM scaffolds are not capable to mimic the mechanical integrity of healthy bone tissues. Manjunath et al. reported PLA scaffolds with a E = 9.98 MPa (SD not available) which was improved to 16.02 MPa (SD not available) with the addition of a PCL matrix.^[^ ^35^, ^38^ ^]^ Depending on the structure (e.g., 0–90° pattern, kagome, or 0–45° or 0–30° pattern) and the presence of ceramic elements, PCL scaffolds displayed compressive strength values between 10 and 30 MPa and *E*_flex_ between 70 and 170 MPa.^[^ ^36^, ^39^, ^40^, ^76^ ^]^ These values are at least one order of magnitude lower than cancellous bone and two orders of magnitude lower than cortical bone. In mandibular bone reconstruction applications, mechanical strength and load‐bearing capacity are crucial factors, as the mandible experiences high loads during its lifespan. However, it is worth noting that low mechanical resistance is not necessarily a concern at the onset of bone regeneration, as patients are not allowed to apply loads to the fracture. Moreover, the implants are usually stabilized by metal reconstruction plates. These devices are shaped on the patient's anatomy and can withstand multiple cyclic masticatory loads, as confirmed by mechanical simulations and tests.^[^ ^78^, ^79^ ^]^ CAD/CAM methods can be used to fabricate the plates from patient scans, resulting in a more accurate shape and improved mechanical properties.^[^ ^78^, ^79^, ^80^ ^]^ For instance, Koper et al. have proposed to use topology optimization, a computational method, to design and fabricate Ti6Al4V SLM plates tailored to the patient's requirements, thereby reducing implant failure.^[^ ^80^ ^]^ As the bone healing process takes place, new bone forms inside the scaffold, increasing its global stiffness. When the healing is complete, the callus is able to sustain the load independently, and the fixation devices can be safely removed.^[^ ^81^ ^]^

## 6 Bioactive Molecules for Mandibular Regeneration

Additionally to the importance of selecting the biomaterials and architectures, to engineer a valuable bone construct, also bioactive molecules (e.g., BMP‐2) are crucial. In fact, they play a pivotal role in correct tissue formation, repair, and homeostasis.^[^ ^82^ ^]^ However, despite the abundant array of choice, growth factors employed for mandibular BTE are almost exclusively confined to one family: BMPs, and in particular BMP‐2.

**Figure 6:**
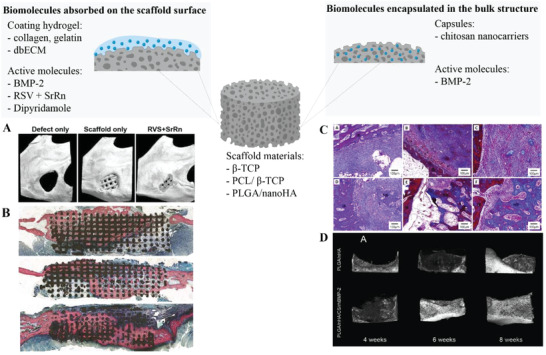
Surface release and bulk encapsulation of growth factors. Created with BioRender.com. A) Micro‐CT images after 8 weeks, for different experimental groups: the empty defect, the defect filled with scaffolds only, and the defect filled with scaffolds loaded with RSV and SrRn. Reproduced with permission.^[^ ^41^ ^]^ Copyright 2020, IOP Publishing. B) Histomorphologic images of the scaffold implanted in the ramus after 8 weeks. Pink = bone, black = residual scaffold, in the control group (above), collagen group (center) and Dipyridamole group (below). Reproduced with permission.^[^ ^37^ ^]^ Copyright 2019, The American Society of Plastic Surgeons. C) Histological images of the bone defect sites at different time‐point. Control group: (A) 4 weeks, (B) 8 weeks, and (C) 12 weeks; Experimental group: (D) 4 weeks, (E) 8 weeks, and (F) 12 weeks. IB = initial bone, NB = new bone. Reproduced with permission.^[^ ^32^ ^]^ Copyright 2019, Elsevier. D) Micro CT reconstruction images of implanted areas at 4, 8, and 12 weeks in the experimental and control groups. Reproduced with permission.^[^ ^32^ ^]^ Copyright 2019, Elsevier.

Mature BMPs are normally secreted from osteoblasts and may either activate their membrane receptors or bind to extracellular matrix (ECM) proteins, such as collagen, and act as a reservoir for neighboring cells.^[^ ^83^ ^]^ These proteins stimulate the differentiation of MSCs into osteoblastic lineage and promote the proliferation of osteoblasts and chondrocytes, being therefore an active player in ossification and bone healing processes.^[^ ^84^ ^]^ For this reason, a series of studies focused on tailoring scaffolds made of bioactive ceramics and/or polymers with recombinant human BMP‐2 (rh‐BMP‐2). The biomolecule can be directly encapsulated in the bulk structure of the scaffold, or first loaded into microcarriers (e.g., hydrogel particles) (**Figure** **6**). Moreover, it can be mixed with hydrogels, such as collagen and demineralized bone ECM (bdECM), to be later casted on the scaffold surface.^[^ ^85^ ^]^ Cao et al. obtained very good results with *β*‐TCP scaffolds coated with an rh‐BMP‐2/gelatin solution, and implanted in primates. The BV/TV ratio obtained through µCT has been reported to increase almost of 20% in the presence of rh‐BMP‐2 (BV/TV% ≈ 85%). Furthermore, cumulative release of rh‐BMP‐2 from scaffolds has been assessed (48.5 ± 6.4% at the 21th day).^[^ ^42^ ^]^ Nokhbatolfoghahaei et al. have also tested *β*‐TCP scaffolds in several conditions in a canine animal model, and always reported higher histomorphometry values of NB in the presence of rh‐BMP‐2 (NB = 48.443 ± 0.250%).^[^ ^39^ ^]^ On the other hand, despite reporting a modest trend of bone formation for PCL/*β*‐TCP scaffolds soaked into a BMP‐2/collagen solution (BV/TV ≈ 13%), results of Lee et al. lacked statistical significance when compared to the control group (PCL/*β*‐TCP alone).^[^ ^36^ ^]^ Similar outcomes were reported in another PCL/*β*‐TCP scaffold study, where intra‐scaffold injections of a rhBMP‐2 and bdECM bioink improved bone‐to‐implant contact ratio (BIC) (BIC = 51.29 ± 14.64%), but NB was again not significantly ameliorated.^[^ ^47^ ^]^ Deviating from the previous works, Deng et al. employed different types of ceramics and polymers than *β*‐TCP and/or PCL, for growth factor delivery. Their PLGA/nanoHA scaffold, complexed with rh‐BMP‐2‐loaded chitosan nanocarriers, allowed considerable tissue formation (BV/TV = 33.7% and NB = 45.5%) in rabbits, and efficient growth factor release (61.38 ± 2.39% on the 30th day) (Figure 6D).^[^ ^32^ ^]^

A valid alternative to natural growth factors for stimulating bone formation within a scaffold is the use of small molecules that can modulate osteogenic cellular pathways. *β*‐TCP scaffolds soaked in a dipyridamole solution exhibited excellent bone growth (26.9 ± 10%) in a rabbit critical‐sized mandibular defect (Figure 6B). Dipyridamole has been proven, indeed, to indirectly activate adenosine A_2A_ receptors that in turn inhibit osteoclasts differentiation and promote osteoblasts differentiation.^[^ ^37^ ^]^ Zhang et al. printed a PCL/TCP/hydrogel mixture filled with two small molecules: RSV and SrRn. RSV and SrRn have several pharmacological effects, like promoting bone formation, osteogenic differentiation and the release of angiogenic factors. The combined use of these two molecules significantly raised BV/TV (≈ 25%) in vivo, compared to the PCL/TCP scaffolds alone (≈10%) (Figure 6A). Cumulative release profiles of RSV and SrRn from the 3D printed scaffolds were also reported (SrRn ≈ 70% and RSV < 30% at day 21).^[^ ^41^ ^]^

BMP‐2 and ‐7 have been approved by the Food and Drug Administration (FDA) for clinical use in open fractures of long bones, non‐unions, and spinal fusion.^[^ ^86^ ^]^ However, growth factors have been reported as the cause of some serious dose‐related side effects, such as exuberant bone growth, osteolysis and ectopic bone formation ^[^ ^37^ ^]^, and it is difficult to find a consensus in the literature regarding the effective dose of BMP‐2 to achieve the expected clinical results.^[^ ^50^ ^]^ Commercially available products recommended a concentration within the milligram per milliliter range (1.05–1.5 mg mL^−1^), but this value represents approximately 200 000 times the estimated physiologic concentration of natural BMP‐2 found in bone.^[^ ^87^ ^]^ Moreover, other drawbacks related to BMPs include short half‐life, protein instability, control over release rate and high production costs, which makes routine application not possible yet.^[^ ^41^ ^]^

From this point of view, small bioactive molecules may represent a valid alternative to growth factors, because they have relatively simple structures, are easy to prepare with consequently  lower batch variability and production cost, and are already employed in a wide variety of biomedical applications.^[^ ^37^, ^41^ ^]^ Most probably, to limit problematics related to FDA approval and uncontrolled side effects on humans, for future applications, one ideal solution would be the development of synthetic materials with intrinsic osteoinductive capacity.^[^ ^88^ ^]^ As mentioned in above sections, differences in chemical composition (calcium phosphate/polymer phase), structural properties (porosity, surface area, grain size, and compressive modulus), and dissolution behavior of a scaffold influence its osteoinductive potential. However, the underlying signal transduction pathways activated by instructive materials are still largely unknown and questions remain to be answered whether this strategy could actually be enough to heal mandibular CSDs, and restore bone tissue at physiological level.

## 7 Vascularization Strategies for Mandibular Regeneration

While incredible advances in terms of material properties, scaffold design and AM technologies have been made, the potential promise of craniomaxillofacial BTE have yet to achieve relevant clinical success. AM scaffolds are currently clinically tested for certain applications, such as the treatment of small defects resulting from enucleation of odontogenic cysts or ridge augmentation. For instance, Naik et al. conducted a case study in 2019 where PCL scaffolds were implanted in 10 patients with small, non‐critical maxilla and mandibular defects. Despite the positive response to the implants, with no pain, swelling or severe inflammation, bone formation within the scaffolds was minimal after 9 months.^[^ ^89^ ^]^ This result could be attributed to the inert nature of the scaffold material. It is important to notice that this case study, as mentioned above, reported the clinical treatment of a small defect.

In vivo, the maximum distance of a cell from its nearest capillary rarely exceeds 200 µm and is usually less than 100 µm, which is why making a 3D construct with a functional vascular network is considered the most crucial challenge in BTE, especially in CSD repair.^[^ ^90^ ^]^ Bone tissue constructs of clinically relevant size (thicker than 1 cm^3^)^[^ ^91^ ^]^ and with metabolic activity resembling that one found in the native tissue (≈10^9^ cell cm^−3^) necessitate to be connected to an active blood flow once grafted in a patient, otherwise necrosis will arise faster than blood vessels infiltration into the scaffold (<1 mm day^−1^).^[^ ^92^ ^]^ Furthermore, a proper level of oxygenation is essential for cellular viability, but it is also important for maintaining cellular functions.^[^ ^93^ ^]^ As a matter of fact, angiogenesis is known to strongly influence osteogenesis. During such processes endothelial cells produce growth factors (e.g., BMP‐2, PDGF) that control the recruitment, proliferation, differentiation, function, and/or survival of various cells including osteoblasts and osteoclasts.^[^ ^94^ ^]^

**Figure 7:**
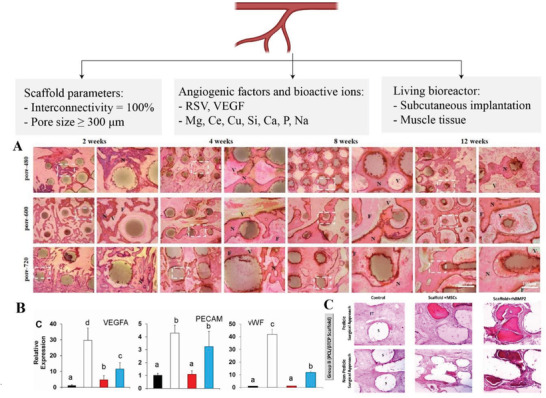
Vascularization strategies for mandible reconstruction. Created with BioRender.com. A) Histological analysis of H/E staining from 2 and 12 weeks, N = new bone, F = fiber, V = vessels. Adapted with permission.^[^ ^44^ ^]^ Copyright 2022, Wiley‐VCH GmbH. B) qPCR analysis of vascular endothelial growth factor A (VEGFA), platelet endothelial cell adhesion molecule (PECAM), and von Willebrand factor (vWF) genes for HUVEC cells. Bars that have different letters are statistically different from each other. Adapted with permission.^[^ ^41^ ^]^ Copyright 2020, IOP Publishing. C) Histological images of H/E‐stained sections from mandibular defects at 12 weeks post‐surgery. Adapted with permission.^[^ ^39^ ^]^ Copyright 2022, Wiley‐VCH GmbH.

In the context of mandible reconstruction, only few strategies have been explicitly explored to boost angiogenesis in vitro and/or in vivo and to promote mass transfer of nutrients and oxygen in the inner core of engineered tissues (**Figure** **7**). A first approach relies on adjusting scaffold key architectural parameters such as porosity, pore size, and interconnection.^[^ ^43^ ^]^ Typically, a pore size ≥ 300 µm is required to facilitate NB and vascularization, while the minimum accepted size seems to be around 100 µm.^[^ ^94^ ^]^ Qin et al. made a comparative analysis of magnesium‐substituted calcium silicate scaffolds having the same porosity (58%) but with different pore sizes (Ø 480, 600, and 720 µm). Histological analysis of rabbit mandibular defects filled with these scaffolds revealed considerably larger blood vessels in the 600 and 720 µm groups compared to the 480 µm group after 12 weeks from implantation (Figure 7A).^[^ ^44^ ^]^ The need of macropores for an efficient vascularization in vivo was also confirmed in a PVA/PU Lay‐Fomm scaffolds study, where growth of a vascular network was prevented because of lack of porosity in the scaffolds used.^[^ ^31^ ^]^ Kang et al. have reported a novel 3D printing technology [integrated tissue–organ printer (ITOP)] to make multi‐material scaffolds with a lattice of microchannels (500 × 300 µm^2^) into human‐scale tissue constructs that are permissive to nutrients and oxygen diffusion. With this technique, a scaffold with human mandible critical defect size (3.6 cm × 3.0 cm × 1.6 cm) made of PCL/TCP and of a composite hydrogel containing hAFSCs was fabricated. After 28 days of culture, osteogenesis was successfully induced in vitro. Unfortunately, ITOP potential was not tested in vivo specifically for mandibular reconstruction.^[^ ^33^ ^]^

A second strategy involves the use of a smart scaffold capable of favoring vessel formation by delivering angiogenic factors to cells in culture. As described in the above section, Zhang et al. proposed 3D printed PCL/TCP composite scaffolds, with a hydrogel‐based bioink, encapsulating bioactive small molecules (RSV and SrRn).^[^ ^41^ ^]^ HUVECs treated in vitro with the released medium of scaffolds loaded with RSV, showed the highest gene expression levels of VEGFA, PECAM‐1, and vWF (Figure 7B).

The most applied scaffold vascularization strategy for mandibular reconstruction consists in exploiting a living bioreactor (i.e., the body) to generate new blood vessels in vivo. Such technique involves two steps: a first implantation of the scaffold within a highly vascularized site of the body (e.g., skeletal muscles) and a subsequent graft of the construct at the bone defect site. Microvascular surgery is also required to anastomose the engineered vessels with those at the recipient site.^[^ ^95^ ^]^ Cao et al. showed the superiority of *β*‐TCP scaffolds cultivated in the latissimus dorsi muscle of primates over a three‐month period.^[^ ^42^ ^]^ Vascularization of the construct was improved due to penetration of host blood vessels from the neighboring muscle tissue and, after orthotopic transplantation in a mandibular defect, pronounced effects of bone regeneration were shown (BV/TV ≈ 85% and NB ≈ 60%). Interestingly, PLGA/TCP composite scaffolds prefabricated in the same manner were resorbed too much and could not be used further. Also Nokhbatolfoghahaei et al. demonstrated a better outcome by using *β*‐TCP scaffolds that preserved a vascularized pedicle from the masseter muscle where they stayed implanted for two months. Significantly greater rate of NB (NB = 48.443 ± 0.250%) was obtained in vivo but, despite all, capillary formation within the constructs could not be proven by histological analysis (Figure 7C).^[^ ^39^ ^]^ Finally, Konopnicki et al. hypothesized that vessels penetration in *β*‐TCP/PCL scaffolds seeded with porcine bone marrow progenitor cells (pBMPCs) was improved by early implanting the constructs in a critical‐size mandibular defect (i.e., before the 14th days of in vitro culture).^[^ ^34^ ^]^

The problem of scaffold vascularization in the context of mandibular BTE remains the narrowest bottleneck that is currently limiting translation of academic research into clinic. Apart from the lack of consistent and well‐documented results, there seems also to be a gap in terms of technical evaluation of an efficient vascularized scaffold. The presence of blood vessels is proven almost exclusively by histological images and expression of pro‐angiogenetic factors. However, it would be suitable to find alternative methodologies able to assess not only the qualitative presence of vessels, but also their functionality, meaning the capability of homogeneously perfusing the engineered construct. Finally, a reliable quantification of the number, structure, hierarchy, and distribution of vessels should always be properly evaluated.

Using the body's own regenerative potential seems an appealing solution to provide scaffolds with well‐formed blood vessels prior implantation in mandibular CSDs. However, this strategy possesses several disadvantages, such as the need of two surgeries, as well as shape and size limitations. In this regard, it would be impossible to regenerate large and articulated bone constructs without excruciating patient discomfort and functional impairment at the ectopic region. A new perspective is therefore of paramount importance. As for the quest of instructive materials to induce bone formation, discovering materials with an intrinsic angiogenetic potential would be desirable also for prompting and guiding vascularization.

A promising route to be better investigated on the mandibular reconstruction topic involves the use of bioactive ions (e.g., Mg, Ce, and Cu) that upon release can lead to increase levels of angiogenic markers.^[^ ^44^, ^94^ ^]^ These ions can be exogenously incorporated in the material's matrix or surface, as it has been done for *β*‐TCP, or they can be constitutively present in the material's chemical structure. The latter category includes silicate glasses (i.e., bioactive glasses), which upon dissolution can release Si, Ca, P and Na ions that were proven to stimulate osteogenesis, neovascularization and/or angiogenesis, and enzymatic activity.^[^ ^96^, ^97^ ^]^ With such instructive materials, not only can the laborious procedure of the living bioreactor strategy be avoided, but many drawbacks related to angiogenic growth factors’ approval from FDA could be bypassed.

As a final remark, in human bones, blood supply is accompanied by innervation that also plays a crucial role in maintaining tissue homeostasis. The maxillofacial region is highly innervated. The mandible is indeed crossed by the inferior alveolar nerve responsible of sensory functions of the tissue.^[^ ^98^ ^]^ When a patient undergoes a mandibular resection surgery part of this nerve is removed, causing a loss of sensation in the lip and chin that can result in impaired functions (e.g., chewing, and speaking). Despite the known importance, innervation seems not to be taken into consideration in BTE approaches for mandibular regeneration. In fact, none of the reviewed studies (Table 1) mentioned an approach for scaffold innervation. Recently, it was demonstrated that the nerve growth factor (NGF) can enhance bone regeneration by improving the repair of sensory nerves in the mandible.^[^ ^99^ ^]^ Moreover, NGF can enhance the activity of osteocytes, promote the differentiation and mineralization of osteoblasts, and vascularization, during the process of implant‐bone binding.^[^ ^100^ ^]^ Ye et al. proposed a biomimetic coating made of NGF‐chondroitin sulfate (CS)‐HA to coat titanium implants, ameliorating early osseointegration, and nerve regeneration in the mandible of beagle dogs.^[^ ^100^ ^]^ Therefore, since osseointegration, vascularization and innervation of the implants are strongly interconnected, more efforts should be placed in this direction in future studies.

## 8 Cell Therapy

**Figure 8:**
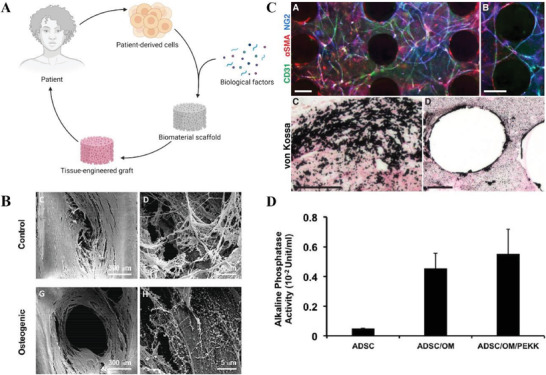
A) Traditional cell therapy approach. Created with BioRender.com. B) SEM images of the seeded scaffolds after 21 days of culture, showing the cells and the matrix filling the pores of the scaffold. Reproduced under the terms of the CC‐BY 4.0 license.^[^ ^31^ ^]^ Copyright 2020, the Authors. Published by Frontiers. C) In vitro vascularization and mineralization of ASC aggregates seeded on PCL scaffolds and cultured for 14 days in vascular (A,B) or osteogenic medium (C,D). Reproduced with permission.^[^ ^48^ ^]^ Copyright 2014, Wiley‐VCH GmbH. D) ALP activity of ADSC aggregates alone, in osteogenic medium (OM) and of aggregates seeded on scaffolds and cultured in culture medium (OM/PEKK). Reproduced with permission.^[^ ^49^ ^]^ Copyright 2017, The American Laryngological, Rhinological and Otological Society, Inc.

Cell therapy is a common approach when trying to repair bone CSDs. In particular, for many years, researchers have been exploring the possibility of using different types of stem cells in combination with scaffolds for BTE. The classical tissue engineering paradigm consists of five steps: harvesting cells from the patient (i); expanding cells in vitro (ii); seeding cells onto a scaffold (iii); maturation of cells with the potential help of suitable growth factors and a bioreactor system (iv); implantation of the functional biological construct into the patient (v) (**Figure** **8**A).

The most appealing cell source is MSCs. These cells have the ability to differentiate into several cell types, including the osteoblastic lineage, and can be harvested from autologous sites (e.g., bone marrow, adipose tissue) so to limit immune response when implanted into the patient.

Among the studies revised, only six followed the traditional tissue engineering approach of seeding cells on scaffolds before the implantation into the animal model. Konopnicki et al. have seeded pBMPCs on PCL/*β*‐TCP scaffolds and the constructs were incubated for 14 days in a rotational oxygen‐permeable bioreactor. After, they were implanted in the defects and compared to unseeded scaffolds and to empty defects. Interestingly, a higher value of PSA was shown for the seeded scaffolds, reaching a value of 22.1 ± 22.45%, compared to the unseeded scaffolds [PSA = 1.87 ± 3.66%].^[^ ^34^ ^]^ Lee et al. treated the defects with PCL/*β*‐TCP scaffolds seeded with ADSCs aggregates (around 350 Hu after 8 weeks, compared to the 250 Hu of the scaffold alone).^[^ ^35^ ^]^ Also in this case, the results suggested that the use of cells seeded on the scaffolds could enhance the mandibular reconstruction. A similar improvement was obtained by Nokhbatolfoghahaei et al. and Park et al., who seeded MSCs and TMSCs on PCL/*β*‐TCP scaffolds, showing quantitatively the improvement in bone regeneration (15.430% ± 0.547% and 5.813% ± 1.345%), compared to scaffolds alone or scaffolds combined only with growth factors.^[^ ^39^, ^40^ ^]^ Finally, Temple et al. and Roskies et al. have proven the efficacy of the seeding of ACSCs aggregates on both PCL (Figure 8C) and PEKK (Figure 8D) scaffolds, showing a higher cell infiltration in the seeded scaffolds after 7 days of in vivo implantation.^[^ ^48^, ^49^ ^]^

An alternative direction to the traditional tissue engineering paradigm is based on the implantation of a cell‐free scaffold to induce tissue growth directly in vivo. As a matter of fact, culturing cells on scaffolds necessitate the help of advanced equipment (e.g., bioreactors, hoods, incubators) and specialized staff. In addition, since stem cell proliferative and differentiation potential is highly dependent on patient age, results may vary or even become ineffective in the elderly. As described in the above sections, it would be ideal to stimulate bone formation only through material intrinsic properties and to exploit the host body regenerative potential.

## 9 In Vivo Studies

Different strategies to improve bone regeneration in vivo have been explored, including the use of cell sources, growth factors, small molecules and prevascularization. However, since experiments have been performed on different animal models, and most importantly, on mandible CSDs of different size, data comparison is not straightforward.

The inhomogeneity of defect size can be attributed to the absence of a clear and quantitative definition of CSD, which has not yet been postulated. This lack of clarity has led to misinterpretation of data and misleading evaluation of tissue engineering approaches. While certain studies have defined a CSD as a segmental defect of a length of 2–2.5 times the diameter of the treated bone, there is still a need for a precise and standardized definition.^[^ ^101^ ^]^ The CSD dimension depends on the animal model, which can be small or large, and on the site of implantation. Small animals include rats, rabbits, mice and guinea pigs, and they are usually used for first steps of in vivo experiments, because of their accessibility, relatively low cost and ethical acceptance.^[^ ^102^ ^]^ Large animal models, on the contrary, are more clinically relevant human models, and they include pigs, dogs, primates and goats/sheep.^[^ ^102^ ^]^ In **Table** **5**, some studies are reported as examples of different size of calvaria defects, varying the animal model.

**Table 5:** Examples of CSD dimensions for different animal models and implantation sites

Animal model	Implantation site	CSD dimension	Ref.
Rabbits	Calvarial	10 mm diameter × 1.2 mm height	[103]
Rodents	Calvarial	4 mm diameter × 2 mm height 8 mm diameter × 0.8 mm height	[104, 105]
Pigs	Calvarial	10 mm diameter × 10 mm height	[106]
Sheep/goats	Tibial^a)^	20 mm length × 21 mm diameter	[107]

^a)^ In general, this animal model is exclusively used for long bone defects.

In this section, we discuss CSD dimensions and volumes in relation to reported values of BV/TV. A parameter called BV/TV**_∆_** has been calculated as the difference between the BV/TV of the experimental (BV/TV_exp_) and the control (BV/TV_ctrl_) group to obtain a value of bone formation comparable among different studies.

**Figure 9:**
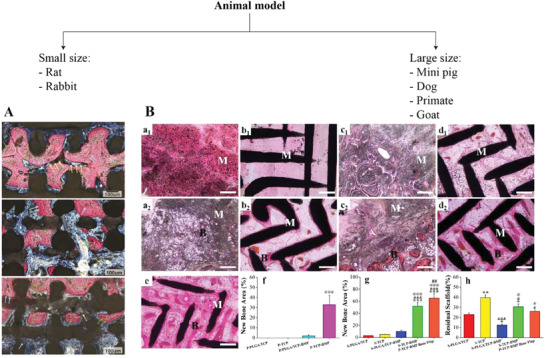
A) Histological evaluation of bone remodelling in Dipyridamole group (Above), Collagen group (Center) and Control group (Below). Vascular structure is depicted as well (yellow arrow). Reproduced with permission.^[^ ^37^ ^]^ Copyright 2019, The American Society of Plastic Surgeons. B) H&E staining after 12 weeks of implantation with P‐PLGA/TCP (a), P‐TCP (b), S‐PLGA/TCP (c), S‐TCP (d), and P‐TCP‐BMP (e) scaffolds, without or with rhBMP‐2 coating. M = materials, B = bone. Percentage of new bone in histological sections obtained from ectopically (f) and orthotopically (g) implanted samples. Percentage of non‐degraded scaffolds (h). Scale bar = 400 µm. Reproduced under the terms of the CC‐BY 4.0 license.^[^ ^42^ ^]^ Copyright 2021, the Author(s). Published by American Chemical Society.

Ten studies (**Table** **6**) adopted a small animal model to test the efficacy of their scaffolds. In general, it is possible to state that defects’ volume of small size animal models was between 6.2 and 750 mm^3^ with smallest linear dimension in the millimeter range (0.5–5 mm), whereas BV/TV**_∆_** spanned from 5% to 15.93% when the CSD of the control group was filled with some material (usually a scaffold without biomolecules nor cells). Finally, it is worth noticing that the duration of the studies is heterogeneous but always in the order of few weeks (usually 6, 8, or 12). In their work, Cooke et al. have shown that BV/TV% was enhanced by 15.93 ± 12.35% in defects filled with Lay‐Fomm scaffolds when compared with the commercial Norian CRS Putty.^[^ ^31^ ^]^ In addition, Zhang et al. have demonstrated a similar increase of bone formation (BV/TV**_∆_** = 14%) in PCL/*β*‐TCP scaffolds loaded with RSV and SrRn with respect to PCL/*β*‐TCP scaffolds alone.^[^ ^41^ ^]^ Even though such values of bone formation are among the highest reported in small size animal model studies, it must be pointed out that the overall scaffold volumes were just 30 (smallest dimension 2 mm) and 9.6 mm^3^ (smallest dimension 1 mm), respectively. In terms of construct viability, contained dimensions is an advantage since oxygen and nutrients diffusional transport to inner cells will be more easily granted. With this in mind, Park et al. and Lopez et al. (**Figure** **9**A) results are therefore more significant, since the authors have still reached comparable BV/TV**_∆_** values (14.32% and 14.60%) but with scaffolds of bigger volumes (400 and 300 mm^3^).^[^ ^37^, ^40^ ^]^ The only study comparing an experimental group (PEEK scaffold seeded with ADSCs) with an empty defect as control group registered, as expected, a quite high BV/TV**_∆_** value (61.27%), being BV/TV_ctrl_ equal to zero.^[^ ^49^ ^]^

**Table 6:** Small animal model studies. Defects’ dimensions and volume correlated with BV/TV_∆_

Animal	Dimensions [mm]	Volume [mm^3^]	BV/TV_exp_ (%)	BV/TV_ctrl_ (%)	BV/TV_∆_ (%)	Time (weeks)	Ref
Rat	Ø_out_ = 5, Ø_in_ = 1.2, *h* = 2.5	46.26	25^a)^	18^a)^	7	8	[30]
Rat	5 × 2 × 3	30	30.26 ± 9.46	14.33 ± 7.94	15.93 ± 12.35	6	[31]
Rabbit	13 × 6 × 4	312	33.7	24^a)^	9.7	12	[32]
Rabbit	10 × 10 × 3	300	26.9 ± 10.7	12.3 ± 8.3	14.60 ± 13.54	8	[37]
Rabbit	10 × 8 × 5	400	53.10; 57.44	43.12	9.98; 14.32	12	[40]
Rat	Ø = 3.5, *h* = 1	9.6	24^a)^	10^a)^	14	8	[41]
Rabbit	10 × 6 × 4	240	23^a)^	18^a)^; 14^a)^	5; 9	12	[44]
Rat	4 × 4 × 2	32	NA	NA	–	1	[48]
Rabbit	15 × 10 × 5	750	61.27 ± 8.24	0	61.27 ± 8.24	20	[49]
Rat	Ø = 4 mm, *h* = 0.5	6.2	17.8 ± 3.77	13.58 ± 6.08	4.22 ± 7.15	4	[51]

^a)^ The exact value was not stated in the paper. NA: data not available.

After all, the ultimate goal of the use of an animal model is to transfer the obtained results to clinical practice. Small size animals drawbacks include limited or rapid cortical remodelling and secondary osteon formation, thinner femoral condyle cartilage, and a cortical bone composition (e.g., hydroxyproline and protein content) differing from that of humans. That is why large animal models (e.g., sheep, goats, pigs, and dogs) are required for demonstrating sufficient translational capacity.

Defect volumes of large size animal model studies under review were in the order of few thousands of millimeters cube, with the lowest value of 400 mm^3^ and the highest of 12 000 mm^3^ (**Table** **7**). Interestingly, the smallest scaffold linear dimension was still in the millimeter range (5–10 mm), just doubling that one of small size animals. This asymmetric conformation still favors mass transport of oxygen by diffusion inside the scaffolds, despite the increment in construct volume of nearly two orders of magnitude. Unfortunately, BV/TV% values were calculated by very few groups. Cao et al. have reported a value of BV/TV**_∆_** of nearly 60% for *β*‐TCP scaffolds loaded with rhBMP‐2, which was further increased to 80% when such scaffold was prefabricated over three months to increase vascularization (Figure 9B).^[^ ^42^ ^]^ Considering the high BV/TV**_∆_** and the large defect size (volume = 3000 mm^3^) this tissue engineering approach can be considered the best among the articles reviewed. On the other hand, Bouyer et al. used a clinical‐grade PLA coated with a polyelectrolyte film loaded with BMP‐2 at 110 (BMP110) and 50 µg cm^−3^ (BMP50) to fill the biggest defect reported (volume = 12 000 mm^3^; smallest dimension = 10 mm).^[^ ^50^ ^]^ Despite the BMP110 group induced an increase in bone volume compared to the control group (autologous bone grafts), the difference (BV/TV_∆_ = 18.33%) was found not to be significant.

**Table 7:** Large animal model studies. Defects’ dimensions and volume correlated with BV/TV_∆_

Animal	Dimensions [mm]	Volume [mm^3^]	BV/TV_exp_ (%)	BV/TV_ctrl_ (%)	BV/TV_∆_ (%)	Time (weeks)	Ref
Mini Pig	20 × 20 × 7	2800	NA	NA	–	8	[34]
Dog	NA	–	NA	NA	–	8	[35]
Dog	20 × 10 × 10	2000	13^a)^; 8^a)^	4^a)^	9; 4	12	[36]
Dog	25 × 10 × 8	2000	NA	NA	–	12	[39]
Primate	20 × 15 × 10	3000	65^a)^;85^a)^	5^a)^	60; 80	12	[42]
Dog	9 × 9 × 10	810	43.79 ± 19.35	45.49 ± 12.09	−1.70 ± 22.82	12	[45]
Mini pig	30 × 24 × 5	3600	NA	NA	–	6	[46]
Dog	NA	NA	NA	NA	–	12	[47]
Mini pig	40 × 30 × 10	12 000	63.33^b)^; 40^b)^	45^b)^	18.33; −5	13	[50]
Goat	40 × 15 × 10	6000	NA	NA	–	24	[53]

^a)^ The exact value was not stated in the paper;

^b)^ Calculated by values of BV and TV reported in the paper. NA: data not available.

The presented results suggest that scaffolds implanted with the addition of cells and/or growth factors performed better in terms of bone formation compared to scaffolds implanted alone, regardless of the animal model and regeneration time. Therefore, although as highlighted above, the use of biological components should ideally be avoided, being an additional element that could delay the approval and commercialization of a scaffold, to date it still appears to be an indispensable component of any tissue engineering strategy.

## 10 Conclusions, Challenges, and Future Perspectives

The mandibular bone tissue presents unique features in terms of origin, function and composition, when compared to other long bones of the human body. Therefore, its regeneration through BTE approaches has to address different requirements. While there are several synthetic substitutes available on the market for small maxillofacial defects, the gold standard for CSDs treatment remains limited to the fibula flap autografts.

AM is a promising technique to fabricate novel implants for CSDs that may serve as alternative treatments. Although multiple AM techniques have emerged, extrusion‐based systems are mostly employed for mandibular BTE applications. It allows creating porous scaffolds, in an easy, accessible and low‐cost way, and with complex geometries, mimicking the micro‐architecture of the mandible. Moreover, a wide range of biomaterials or composites can be processed to approach the native mandible composition, ensuring the osteo‐inductive and ‐conductive properties. Specifically, for mandibular BTE, the most used materials are composites made of *β*‐TCP and PCL. Although scaffolds mechanics can be tuned by composite selection or geometry, current scaffolds are not able to withstand the high mechanical loading of the jaw. To ensure this stability in the early healing phase, metal reconstruction plates have to be co‐implanted. AM scaffolds can be integrated with growth factors and biomolecules (both natural and synthetic ones) to enhance osteo‐ and angiogenic differentiation of resident stem cells. To stimulate osteoblastic differentiation, BMP‐2 is most comply used as biomolecule. However, the use of BMPs is associated with dose‐related side effects, pushing research toward controlled release strategies, such as the use of hydrogels, carriers or coatings, integrated in the scaffolds. Moreover, scaffolds can be pre‐seeded with cells. The most commonly used cellular components are MSCs, thanks to their potential to differentiate into osteoblasts. They can be harvested from the patient itself, to use them as an autologous component in the synthetic scaffolds.

In the review, we specifically investigated the effect of scaffolds on in vivo new bone formation, comparing different CSD dimensions and animal models. The results suggested that the combination of scaffold materials, and growth factor and/or pre‐seeded cells is the most effective approach in terms of bone formation.

Regardless, nowadays, significant challenges are limiting the translation of the constructs into the clinic. First, the main limitation is the lack of vascularization within the scaffolds, which induces necrosis due to absence of nutrients and oxygen. Here, the best approach seems to be the pre‐vascularization of the constructs. However, this practice is time demanding and surgically complex, requiring the connection between the newly formed vessels and the natural ones. Second, as previously mentioned, the innervation of the constructs is an aspect that has not yet been investigated. Therefore, in this area more extensive research is required. Lastly, this review has shown the lack of a quantitative and precise definition of what is to be considered a CSD, resulting in inhomogeneity in the methodological approaches in the testing of scaffolds in vivo, and in difficult and misleading evaluation of the different tissue engineering approaches. Therefore, it would be recommended to establish standard guidelines, with a defined size for various animal models and defect sites. In conclusion, although research on AM scaffolds, integrated with bioactive molecules and cell therapy, has made many advances in the last decades, there is still room for improvement in next‐generation scaffolds.

## Conflict of Interest

The authors declare no conflict of interest.
